# No Evidence of Perceptual Pseudoneglect in Alexithymia

**DOI:** 10.3390/brainsci11030376

**Published:** 2021-03-15

**Authors:** Carmelo Mario Vicario, Gabriella Martino, Alex Marcuzzo, Giuseppe Craparo

**Affiliations:** 1 Dipartimento di Scienze Cognitive, Psicologiche, Pedagogiche e Degli Studi Culturali (COSPECS), Università di Messina, 98121 Messina, Italy; alexmarcuzzo98@gmail.com; 2 Department of Clinical and Experimental Medicine, University of Messina, 98122 Messina, Italy; gabriella.martino@unime.it; 3 Faculty of Human and Social Sciences, UKE-Kore University of Enna, Cittadella Universitaria, 94100 Enna, Italy; giuseppe.craparo@unikore.it

**Keywords:** alexithymia, right hemisphere, line bisection, pseudoneglect

## Abstract

Neuroscience research links alexithymia, the difficulty in identifying and describing feelings and emotions, with left hemisphere dominance and/or right hemisphere deficit. To provide behavioral evidence for this neuroscientific hypothesis, we explored the relationship between alexithymia and performance in a line bisection task, a standard method for evaluating visuospatial processing in relation to right hemisphere functioning. We enrolled 222 healthy participants who completed a version of the 20-item Toronto Alexithymia Scale (TAS-20), which measures alexithymia, and were asked to mark (bisect) the center of a 10-cm horizontal segment. The results document a significant rightward shift in the center of the line in participants with borderline and manifest alexithymia compared with non-alexithymic individuals. The higher the TAS-20 score, the greater the rightward shift in the line bisection task. This finding supports the right hemisphere deficit hypothesis in alexithymia and suggests that visuospatial abnormalities may be an important component of this mental condition.

## 1. Introduction

Alexithymia is a stable personality characteristic [1,2] characterized by a disturbance of affective-emotional processing, which causes difficulties in verbally identifying and describing feelings and emotions [3]. Research in the field reports an alexithymia prevalence rate of 13% in the non-clinical population, where the alexithymia rate appears to be nearly double in men (17%) compared with women (10%) [4]. In terms of impact, alexithymia may be associated with somatic sensations that accompany emotional arousal and may be related to somatic diseases such as inflammatory bowel disease and type 2 diabetes mellitus [5,6]. It is frequently associated with various psychopathological conditions such as depression [7], anxiety [8], and addiction [9], where the prevalence rate is higher than 30% [10].

Research in cognitive neuroscience has provided a brain basis for this affective deficit, showing that alexithymia can be characterized by left hemisphere dominance and/or right hemisphere deficit [11]. Neuroimaging investigations have shown a broad network of cortical and subcortical regions, including regions that are outside the canonical circuit of emotion processing.

This is the case for the parietal cortex, a cortical region involved in several cognitive abilities including spatial attention [12] and magnitude processing [13,14,15]. For example, Kano et al. [16] reported that alexithymics show less activation in the right inferior parietal cortex (and right prefrontal cortex) and greater activation in the left inferior parietal cortex compared with non-alexithymic individuals. A negative correlation was found between the severity of alexithymia and the activation of the right inferior parietal lobe [17]. More recently, Imperatori et al. [18] found a decrease in alpha connectivity between the right parietal lobe and the right temporal lobe.

In the literature, a lesion in the right inferior-parietal cortex was reported to be frequently associated with hemispatial neglect [19], a syndrome characterized by reduced or absent awareness for the contralesional space (i.e., the left spatial side) and an attentional bias to the right side. Similar deficits in spatial processing were found for lesions in the right frontal lobe (e.g., [20]).

An ideal test for assessing the visuospatial and attentional deficits described above [21] is the line bisection task. Over the years, this task has become increasingly popular as a diagnostic tool due to its ease of use and the relative ease with which results are interpreted [22]. The line bisection task requires participants to identify and mark the center of a straight line printed on a piece of paper. Patients with a lesion in the right hemisphere, such as patients with hemispatial neglect, identify and mark the center of the segment in the direction of the damaged hemisphere (i.e., the right hemisphere) [23].

In contrast to this spatial attention bias to the right side, neurologically normal individuals generally shift to the left of the true center when dividing horizontal lines [24]. This behavioral phenomenon, called pseudoneglect [25], has been associated with a dominant right hemisphere, which includes regions of the parietal and frontal lobes such as the intraparietal sulcus [26] and the inferior frontal gyrus [27].

Given the close relationship between the activation level of the right fronto-parietal network, the performance in the line bisection task, and evidence of the hypoactivation of the right fronto-parietal cortex in alexithymia, we aimed to test whether this personality trait [1] predicts spatial representation in healthy individuals. In agreement with the right hemisphere deficit hypothesis in alexithymia [11], we predicted a reduced or even absent pseudoneglect in individuals with this mental condition.

## 2. Participants

In total, 225 healthy participants (college students) participated in this study (113 women, age range between 18 and 30 years). The average age was 23.58 years ± 2.98 standard deviation (*SD*). All participants provided their written informed consent prior to inclusion in the study and were naïve about its purpose. The data were anonymously collected. The study was approved by the local ethics board (COSPECS Department, approval code: COSPECS_10_2020) and was conducted in agreement with the principles of the Declaration of Helsinki.

## 3. Materials and Measures

### 3.1. The 20-Item Toronto Alexithymia Scale (TAS-20)

The TAS-20 is a self-report scale used to measure alexithymia. It is composed of three subscales: (a) difficulty with identifying feelings, (b) difficulty with communicating feelings to others, and (c) external-oriented thought. Bagby et al. (1994) [28] proposed three cut-off scores for the classification of individuals: alexithymic subjects (≥61), borderline (score range between 51 to 60), and non-alexithymic subjects (≤50). In this study, the Italian version of the Toronto Alexithymia Scale was used, which was validated by Bressi et al. [29] (1996, Cronbach’s alpha: 0.75).

### 3.2. Line Bisection Task

Participants were asked to sit in a chair and use their preferred hand to mark the center of a single 10-cm horizontal line printed on a A4 sheet with a pen. The sheet was placed on a desk in front of the participant. They were asked to bisect the line within a few seconds, and were not allowed to move the sheet at will. The line bisection task was always performed after the completion of the TAS-20. Participants were tested in a quiet room located in the Faculty of Human and Social Sciences, UKE-Kore University of Enna, and in the department of Cognitive Sciences, University of Messina. Scores were measured manually. Pseudoneglect was indicated by line bisection scores less than 5 cm.

### 3.3. Data Analysis

Statistical analysis was performed using STATISTICA (StatSoft. Inc., Tulsa, OK, USA) version 8.0. Data were entered into a one-way ANOVA to identify differences between the three groups (alexithymic, borderline, and non-alexithymic) in bisection line performance and demographic variables. Student’s *t*-tests (Bonferroni corrected) were conducted in the case of significant ANOVA results. Additional Student’s *t*-tests were performed to verify significant shifts from the real center of the presented segment. Finally, Pearson’s correlation analysis was performed to investigate the association between TAS-20 scores and bisection line performance. For all analyses, the statistical significance level was set to *p <* 0.05.

## 4. Results

**Figure 1:**
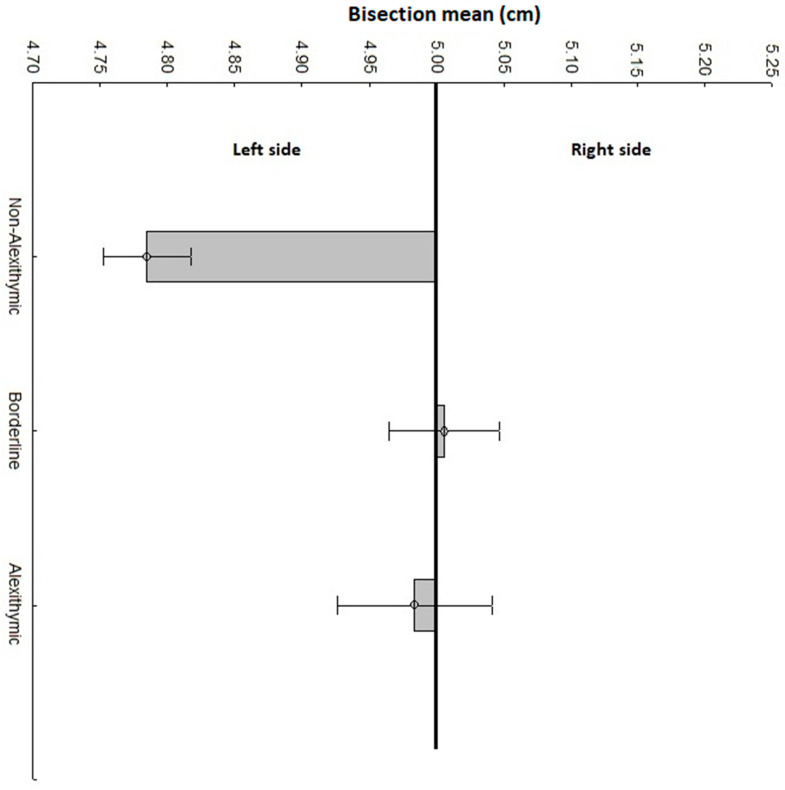
Performance in the line bisection task of alexithymic, borderline, and non-alexithymic participants. Deviations from the central vertical line indicate the mean bisection shift of the three groups of participants for the 10-cm segment. Scores less than 5 cm indicate a deviation to the left (i.e., pseudoneglect). Scores greater than 5 cm indicate a deviation to the right. Vertical bars indicate standard error.

Three participants were removed from the final analysis as their bisection line performance was ±3 *SD* from the average. Our final sample was composed of *N* = 37 alexithymic (TAS-20 score: *M* = 66.48 ± 0.927), *N* = 72 borderline (TAS-20 score: *M* = 55.44 ± 0.664), and *N* = 113 non-alexithymic (TAS-20 score: *M* = 39.29 ± 0.927) participants. The TAS-20 score was statistically different between the three groups (*F*(2, 219) = 392, *p <* 0.001, η_p_^2^ = 0.781). No significant difference was reported for the sex (*F*(2, 219) = 1.99, *p* = 0.138) or age (*F*(2, 219) = 0.403, *p* = 0.668) variables between the three groups. The ANOVA documented a significant group difference in bisection line performance (*F*(2, 219) = 10.46, *p <* 0.001, η_p_^2^ = 0.087). Post-hoc comparison documented a lower line bisection score in non-alexithymic participants (*M* = 4.784 ± 0.032 cm) compared with borderline (*M* = 5.005 ± 0.041 cm, *p <* 0.001) and alexithymic (*M* = 4.983 ± 0.057 cm, *p* = 0.008) participants. No difference was identified between borderline and alexithymic participants (*p* = 1.000). See Figure 1 for details. One sample *t*-test against the real center of the segment (i.e., 5 cm) documented a significant difference for non-alexithymic participants (t(112) = −6.060, *p <* 0.001). This result confirms pseudoneglect in non-alexithymic participants. On the other hand, no difference was reported for borderline (t(71) = 0.146, *p* = 0.883) or alexithymic (t(36) = −0.325, *p* = 0.756) participants.

**Figure 2:**
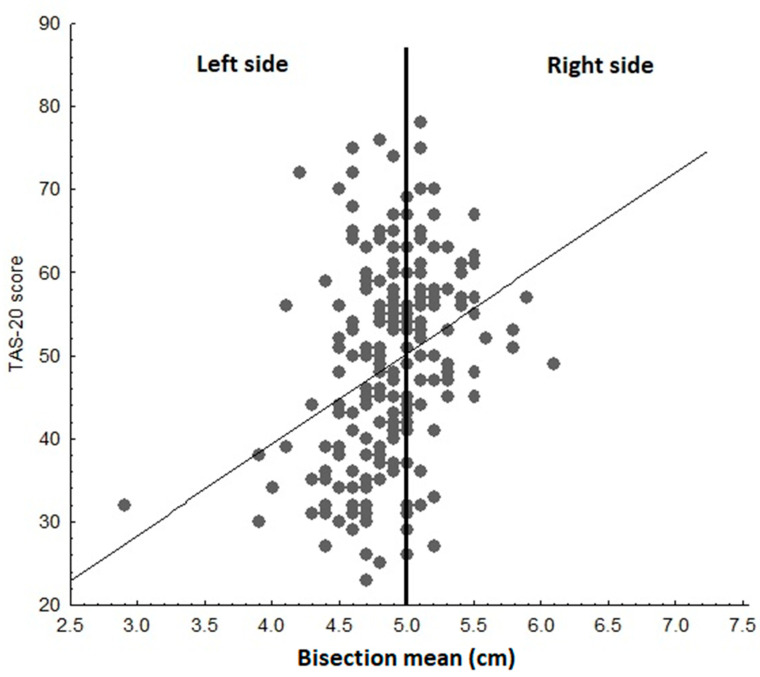
A plot of the 20-item Toronto Alexithymia Scale (TAS-20) scores and line bisection performance of all participants. The figure shows a positive correlation between TAS-20 scores and rightward bias.

Correlation analysis provided further support to the ANOVA results. We found a positive correlation between TAS-20 and line bisection scores (*r* = 0.329, *p <* 0.001). Therefore, the higher the TAS-20 score, the larger the rightward shift in the line bisection task (Figure 2).

## 5. Discussion

Motivated by neuroimaging evidence [16,17] of reduced activation in the right fronto-parietal network in alexithymia, here, we used the line bisection task to provide behavioral evidence to this functional asymmetry. As expected, the results of our sub-group of non-alexithymic participants replicate the pseudoneglect bias (i.e., leftward shift in line bisection) reported in previous studies on neurologically normal individuals [24]. We also confirmed our research hypothesis of no pseudoneglect in participants classified as overtly alexithymic compared with non-alexithymic participants. Interestingly, the absence of pseudoneglect was also confirmed for participants with borderline TAS-20 scores. This result is also corroborated by the overall positive correlation between TAS-20 and rightward bias. Therefore, the greater the severity of the alexithymia trait, the greater the tendency to mark the center on the right side of the presented segment.

Overall, our results are in line with neuroimaging evidence of reduced right fronto-parietal activation in this mental condition and, more generally, with models proposing a left hemisphere dominance and/or a right hemisphere deficit [11] in alexithymia.

Evidence of reduced or even absent pseudoneglect in alexithymia provides new insights into understanding this mental condition, as it suggests that this personality trait is also characterized by non-emotional features. It adds new evidence to the results of altered cognitive processing in alexithymia [30] by documenting an abnormal representation of space in this mental condition. 

Although we investigated the relationship between alexithymia and line bisection in healthy participants, the results of our study may be useful for the interpretation of altered pseudoneglect (i.e., rightward bias) in psychopathology and mental illnesses such as schizophrenia and attention deficit hyperactivity disorder (ADHD) [31]. Schizophrenia is a severe psychiatric illness frequently associated with alexithymia [32] and there is evidence of a link between alexithymia and ADHD [33]. Evidence of a rightward bias in healthy individuals with borderline/manifest alexithymia in the absence of psychiatric conditions suggests that the origin of the rightward shift in the clinical populations mentioned above (i.e., schizophrenia and ADHD) may be linked, at least in part, with this personality trait. This could question the suggestion that an alteration in pseudoneglect should be considered an endophenotype of schizophrenia [34]. However, this question remains to be investigated, as no research has explored the mediating role of the alexithymia trait in the line bisection performance of the aforementioned clinical populations. Further investigation in the field is needed to explore this hypothesis in dedicated studies.

In conclusion, this is the first evidence of abnormal spatial representation in individuals with borderline and manifest alexithymia. In future investigations, it would be interesting to address the role of handedness, which was not considered in the present study. It would also be important to explore the existence of any dissociation between near and far space in the line bisection task, given the evidence that distinct neural networks are involved in the bisection of lines placed in the near and far space (i.e., the dorsal stream for the bisection of lines placed in the near space and the ventral stream for the bisection of lines placed in the far space [35]. 

Finally, given the close relationship between space and numbers [36,37,38,39], as well as between space and time [40,41,42,43], it would be intriguing to explore the bisection of time and numbers in alexithymia, as a pseudoneglect-like effect for this information has been documented in healthy individuals [44,45].

## Data Availability

Data access is granted by forwarding a request to the corresponding author.
